# Odorant Binding Proteins in *Tribolium castaneum*: Functional Diversity and Emerging Applications

**DOI:** 10.3390/insects16121250

**Published:** 2025-12-10

**Authors:** Lei Wang, Yujie Lu, Zongpei Zhao

**Affiliations:** 1 School of Grain Science and Technology, Jiangsu University of Science and Technology, Zhenjiang 212100, China; wanglei-best@just.edu.cn; 2 Jiangsu Provincial Engineering Research Center of Grain Bioprocessing, Zhenjiang 212100, China

**Keywords:** *Tribolium castaneum*, odorant binding proteins (OBPs), molecular structure, gene expression, pest control, comparative biology, research trends, detoxification, signal transduction, structural biology

## Abstract

*Tribolium castaneum*, the red flour beetle, is both a major pest of stored products and a valuable genetic model. Odorant Binding Proteins (OBPs), traditionally associated with olfaction, are now recognized as multifunctional, contributing to detoxification, immunity, and reproduction. This review synthesizes recent advances in the molecular structure, gene expression, and functional characterization of *T. castaneum* OBPs—particularly TcOBPC11, TcOBPC12, TcOBPC17, and TcOBP7G. Experimental evidence, including RNA interference, ligand-binding assays, and expression profiling, supports their role in defense against xenobiotics. Comparative genomic analyses reveal lineage-specific expansions and adaptive evolution, especially in Minus-C OBPs. Translational applications include RNA interference (RNAi)-based pest control, OBP-targeting repellents, and biosensors. Outstanding challenges remain, including structural resolution and functional redundancy. Future research integrating CRISPR, single-cell transcriptomics, and structural biology will be critical to decode OBP regulatory networks and leverage their potential in pest management and biotechnology.

## Simple Summary

Stored product loss threatens food security worldwide. This review examines odorant binding proteins in the red flour beetle, a major pest and genetic model. We show how these small proteins, once viewed only as smell carriers, also act in chemical defense, detoxification, immunity, and reproduction. Evidence from tissue- and stage-specific expression maps, gene-silencing experiments, and ligand-binding assays supports roles for specific family members—C11, C12, C17, 7G, and the 9A/9B pair—that respond to diverse harmful chemicals. Comparative analyses reveal lineage-specific expansions and adaptive change, with rapid diversification in a subgroup lacking two conserved bonding sites. Translational opportunities include gene-silencing baits, behavior-modifying chemicals that disrupt binding, and sensors that harness protein–odor interactions. Key barriers remain, notably the shortage of high-resolution three-dimensional structures and functional overlap among related proteins. Priorities include stable genome editing to create single and multiple knockouts, single-cell maps to assign functions, and structural methods to visualize binding and release. Integrating these advances into system-level models can deliver precise, environmentally responsible protection of stored foods while reducing broad insecticide use.

## 1. Introduction

### 1.1. Tribolium castaneum: A Global Pest and Powerful Genetic Model

*Tribolium castaneum* (Herbst), the red flour beetle, holds a dual identity in the scientific world. On one hand, it is a cosmopolitan and economically significant pest of stored agricultural products, including grains, flour, cereals, and processed foods. Its infestations lead to substantial losses through direct consumption and contamination with metabolic byproducts, such as quinones, which render food unpalatable and potentially harmful [1,2]. On the other hand, *T. castaneum* has emerged as a premier non-Drosophila model organism for a wide spectrum of biological research [3]. Its utility is underpinned by several advantageous characteristics: it is easy and inexpensive to rear in the laboratory, has a short generation time of approximately one month, and, most critically, possesses a high-quality, fully sequenced genome [4]. The Tcas5.2 reference genome assembly (GenBank: GCF_000002335.3), along with recent annotation enhancements, provides a robust framework for genetic and genomic investigations [5].

A particularly powerful feature of *T. castaneum* is its remarkable susceptibility to systemic RNA interference (RNAi) [4]. This allows for efficient and targeted gene knockdown, making it an exemplary system for functional genomics to dissect gene function and identify potential targets for novel pest control strategies [6]. These attributes have facilitated in-depth studies into fundamental insect biology, including development, insecticide resistance, and, central to this review, the mechanisms of chemosensation [2].

### 1.2. Odorant Binding Proteins: Canonical Roles in Insect Chemoreception

The ability of insects to navigate their environment, find food, locate mates, and avoid threats is profoundly dependent on their sophisticated chemosensory system [7]. At the forefront of this system are the Odorant Binding Proteins (OBPs), a diverse family of small (typically 10–30 kDa), soluble, and highly abundant proteins found in the aqueous sensillar lymph of chemosensory organs, primarily the antennae and mouthparts [8]. The canonical model of OBP function posits that they perform the crucial initial step in olfaction: capturing hydrophobic semiochemicals—such as host plant volatiles, pheromones, and kairomones—and solubilizing them for transport across the hydrophilic lymph. This OBP-odorant complex is then delivered to membrane-bound olfactory receptors (ORs) situated on the dendrites of olfactory sensory neurons, triggering a signal transduction cascade that results in an electrical signal [9].

**Figure 1:**
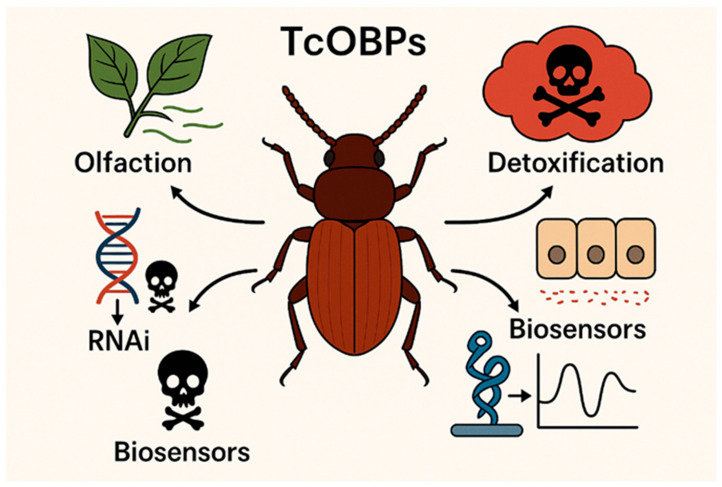
*Tribolium castaneum* as a Dual Model and the Expanded Functions of OBPs Beyond Olfaction.

Insect OBPs are structurally classified into several subfamilies based on the number and pattern of their conserved cysteine residues, which form intramolecular disulfide bridges essential for protein stability. The main subfamilies include “Classic” OBPs, which possess six conserved cysteines; “Plus-C” OBPs, which have additional cysteines and a conserved proline residue; and “Minus-C” OBPs, which are characterized by the loss of the second and fifth ancestral cysteines (C2 and C5) [10]. While this classification provides a structural framework, the functional landscape of OBPs is proving to be far more complex than originally envisioned. A central theme of this review is the paradigm shift from viewing OBPs solely as passive odorant carriers to recognizing their active roles in a multitude of physiological processes, including detoxification of xenobiotics and innate immunity, functions that are particularly relevant for a generalist pest like *T. castaneum* (Figure 1) [11].

## 2. Molecular Architecture and Functional Dynamics of *T. castaneum* OBPs

### 2.1. Structural Hallmarks and Subfamily Diversity

The fundamental architecture of insect OBPs, including those in *T. castaneum*, is a compact, globular fold predominantly composed of six to seven α-helices [12]. This structure is stabilized by a network of intramolecular disulfide bridges, which are the basis for OBP classification. In Classic OBPs, three disulfide bonds (C1-C3, C2-C5, C4-C6) create a stable scaffold that cradles a hydrophobic internal binding pocket, designed to sequester lipophilic odorants. Variations in this pattern, such as the absence of the C2-C5 bridge in Minus-C OBPs, can lead to increased structural flexibility and altered ligand-binding characteristics [13].

While experimentally determined three-dimensional structures for most *T. castaneum* OBPs (TcOBPs) remain elusive, this gap has spurred the proficient use of computational biology to generate valuable structural hypotheses [14]. Homology modeling, which builds a structural model of a target protein based on the known structure of a related protein, has been pivotal. For instance, the homology model of TcOBPC12 references available structures/models of related beetle OBPs and indicates a binding pocket composed of both hydrophobic and polar residues, consistent with interactions with phenolic ligands such as eugenol [15]. Similarly, modeling of the Minus-C OBP TcOBP7G provided insights into how the absence of a disulfide bond might alter its binding pocket geometry and ligand preferences [16]. The recent advent of highly accurate protein structure prediction tools, such as AlphaFold 2, is set to revolutionize this field, providing high-confidence models that can accelerate structure-based functional studies and rational ligand design [17].

However, it is important to emphasize that these TcOBP models still represent structural hypotheses rather than experimentally validated structures. Sequence identity with available OBP templates is moderate, and the most variable regions—loops and termini that often shape the entrance and fine geometry of the binding pocket—are also the least reliable in homology models. As a consequence, detailed predictions about side-chain orientations and specific ligand contacts should be interpreted with caution and ideally confirmed by site-directed mutagenesis and high-resolution structural methods.

**Figure 2:**
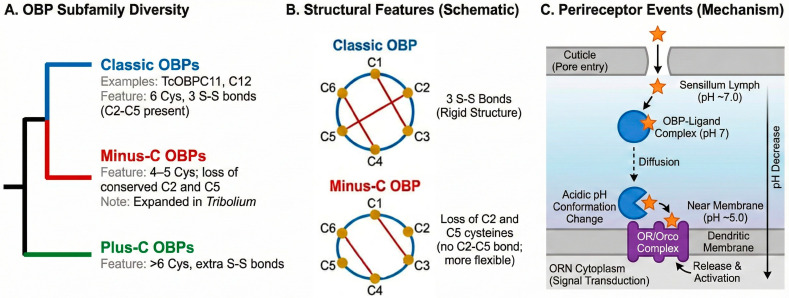
Overview of OBP Diversity, Structural Features, and Functional Mechanism. (**A**) Phylogenetic diversity showing the main OBP subfamilies. Classic OBPs typically have 6 conserved cysteines. Minus-C OBPs (expanded in *Tribolium*) are characterized by the loss of the conserved C2 and C5 cysteines. Plus-C OBPs carry additional cysteines. (**B**) Schematic comparison of Classic vs. Minus-C OBP structures. Classic OBPs possess 6 cysteines forming 3 rigid disulfide bonds (red lines). Minus-C OBPs have lost the ancestral C2 and C5 cysteines, and therefore lack the C2–C5 disulfide bond, resulting in a potentially more flexible structure (dashed outline). (**C**) Schematic model of perireceptor events. Hydrophobic odorants (orange stars) bind to OBPs at neutral pH and are released to the Olfactory Receptor (OR) complex in the acidic environment near the dendritic membrane.

Beyond static structures, molecular dynamics simulations have highlighted the importance of protein flexibility. These studies suggest that the conformational dynamics of loops connecting the α-helices, as well as a flexible C-terminal region, are critical for accommodating a diverse range of ligands and for the process of ligand entry and release. This inherent flexibility likely underpins the ability of a limited number of OBPs to interact with a vast chemical landscape [18]. These structural themes and subfamily relationships, together with a schematic view of perireceptor events, are summarized in Figure 2.

### 2.2. Ligand Binding: Specificity, Affinity, and Release Mechanisms

A major focus of recent research has been the functional characterization of TcOBP ligand-binding profiles, which has been instrumental in uncovering their diverse physiological roles. These studies are predominantly conducted using in vitro fluorescence competitive binding assays, where a fluorescent probe, such as N-phenyl-1-naphthylamine (1-NPN), binds to the OBP’s hydrophobic pocket. The ability of a test ligand to displace this probe is measured, allowing for the calculation of binding affinity (dissociation constant, Kd, or inhibition constant, Ki) [19].

These assays have revealed distinct binding specificities that align with the beetle’s ecology. Several TcOBPs are clearly implicated in defense and detoxification. TcOBPC12 binds the plant-derived compound eugenol; reported affinities vary across assays, with estimates in the low micromolar range [14]. Its role in responding to various chemical agents was further supported by subsequent studies. Likewise, TcOBPC11 and TcOBPC17 bind components of plant essential oils, such as those from *Artemisia vulgaris* [20,21]. More recently, TcOBPC02 has been suggested to participate in the beetle’s defense against eucalyptol, although direct experimental validation remains limited [22]. In contrast, other OBPs have more canonical olfactory roles. The antennally expressed TcOBP9A and TcOBP9B are implicated in sensing broad food-related volatiles, including green leaf volatiles; current evidence is primarily from expression patterns and functional inference, suggesting a role in general food-source recognition rather than specific pheromone detection [23].

A critical and still-debated aspect of OBP function is the mechanism of ligand release at the surface of the neuronal dendrite. The leading hypothesis is a pH-dependent conformational change [24]. The sensillar lymph is maintained at a generally neutral pH, while the microenvironment near the negatively charged dendritic membrane is thought to be more acidic. This pH drop is proposed to induce a conformational change in the OBP, particularly in the C-terminal region, which “unplugs” the binding pocket and lowers the affinity for the ligand, facilitating its release to the receptor [25]. This model is supported by biophysical evidence for TcOBPs. Circular dichroism (CD) spectroscopy has demonstrated that both TcOBPC12 and TcOBP9A undergo significant conformational changes, with a decrease in α-helical content, as the pH drops from 7.4 to 5.0. This change is accompanied by a marked reduction in ligand-binding affinity, providing strong evidence for this release mechanism in *T. castaneum* [14,21,23]. In addition to pH-driven conformational changes, several alternative or complementary mechanisms have been proposed based on work in other insects. These include allosteric gating triggered by direct interactions with olfactory receptors or membrane lipids, OBP multimerization, and the participation of auxiliary proteins such as sensory neuron membrane proteins (SNMPs). Although such mechanisms have not yet been dissected in detail in *T. castaneum*, they highlight that pH-dependent release is unlikely to be the only solution and that OBP–receptor coupling probably involves a broader set of dynamic molecular events.

At the same time, fluorescence competitive binding assays using 1-NPN have inherent limitations. They report relative affinity under a defined set of in vitro conditions rather than absolute specificity under physiological conditions, and are sensitive to factors such as inner-filter effects, probe solubility, and pH-dependent changes in protein conformation. Consequently, high apparent affinity in these assays does not automatically translate into in vivo functional relevance, and binding data are best interpreted in combination with expression profiles and genetic evidence such as RNAi phenotypes.

### 2.3. The OBP-Receptor Interface and Signal Transduction

The final step in the canonical OBP pathway involves the delivery of the odorant to a membrane-bound receptor complex, which converts the chemical signal into an electrical one. In insects, this is typically a heteromeric complex formed by a specific, ligand-binding Odorant Receptor (OR) and a highly conserved coreceptor known as Orco [26,27]. In addition to OR–Orco complexes, insects also deploy ionotropic receptors (IRs) and gustatory receptors (GRs) for chemosensory detection, and it is conceivable that soluble binding proteins contribute to ligand delivery or modulation across these receptor classes as well, although such roles remain largely unexplored in *T. castaneum*.

*T. castaneum* possesses a remarkably large repertoire of over 250 predicted OR genes, reflecting the chemical complexity of its environment [28]. The Orco protein is essential for the proper trafficking, membrane localization, stability, and ion channel function of the entire OR-Orco complex [27,29,30]. Studies in other beetles have shown that silencing Orco via RNAi effectively disables odorant sensing, underscoring its central role [31].

While direct evidence for specific OBP-OR interactions in vivo remains limited, it has been hypothesized that general odorant binders like TcOBP9A may interact with certain Ors, such as TcOR16 and TcOR18, based on co-expression and response profiles [23,32,33]. In addition, preliminary evidence suggests that TcOBPC12 may colocalize with TcOrco in sensory neurons under chemical stress conditions, although further validation is needed to confirm a functional OBP-receptor coupling [21,22]. The olfactory system is further complicated by the presence of other protein families, such as SNMPs, which may also participate in the receptor complex, particularly for pheromone detection in some insects [34].

## 3. Genomic Organization, Expression, and Regulation

### 3.1. A Spatiotemporal Expression Atlas of T. castaneum OBPs

The functional diversity of the OBP family is mirrored by the remarkable specificity of their gene expression patterns across different tissues, developmental stages, and sexes. This spatiotemporal regulation provides strong clues about the physiological roles of individual OBPs, as summarized in Table 1. A key finding is that many TcOBPs are not confined to the antennae. For instance, TcOBPC11, TcOBPC17, and the recently characterized TcOBPC02 are all highly expressed in tissues central to metabolism and detoxification, such as the fat body, Malpighian tubules, epidermis, and hemolymph [35]. This expression pattern strongly supports their proposed roles in defense against xenobiotics, which are processed in these organs. In contrast, other OBPs like TcOBP9A and TcOBP9B show classic antennal-specific expression, consistent with a primary role in olfaction [23].

Expression levels are also dynamically regulated throughout the beetle’s life cycle. For example, TcOBPC11 expression peaks during the late larval and adult stages, while TcOBPC02 is most abundant in early larvae and pupae [22]. This temporal orchestration likely reflects the different chemical challenges and physiological requirements of each developmental stage. Furthermore, sexually dimorphic expression points to roles in reproduction.

TcOBP10 is reported to show male-biased expression in the antennae, suggesting a possible role in detecting sex pheromones. TcOBP7G has been detected in female reproductive tissues and may be associated with oviposition, but direct functional validation is still lacking [16].

Our ability to map these expression patterns has been greatly enhanced by methodological advances. While quantitative real-time PCR (qRT-PCR) and RNA sequencing (RNA-Seq) provide quantitative data, techniques like fluorescent in situ hybridization (FISH) and, more recently, single-cell RNA sequencing (scRNA-seq) offer unprecedented spatial resolution. For example, FISH revealed that the closely related and genomic-linked TcOBP9A and TcOBP9B are, remarkably, expressed in mutually exclusive, non-overlapping populations of sensory neurons within the antenna [23]. Although scRNA-seq datasets for *T. castaneum* remain limited, preliminary studies suggest distinct clusters of sensory neurons may express unique combinations of OBPs and ORs [36].

### 3.2. The Regulatory Network: Transcriptional, Hormonal, and Environmental Control

The specific expression patterns of OBP genes are orchestrated by a complex regulatory network that integrates diverse internal and external signals. A dominant theme in *T. castaneum* research is the induction of OBP expression by chemical cues. Exposure to plant secondary metabolites, such as components of essential oils like eugenol and eucalyptol, triggers a significant upregulation of defense-related OBPs, including TcOBPC11, TcOBPC12, TcOBPC17, and TcOBPC02 [20,21,35]. This response is not limited to natural compounds; sublethal doses of synthetic insecticides also induce these same genes, providing a direct link between OBP expression and insecticide resistance mechanisms [9].

The molecular basis for this induction is beginning to be understood. Although functional data are limited, promoter analysis of the TcOBPC12 gene has identified binding sites for well-known stress-responsive transcription factors, including Nuclear Factor Erythroid 2-Related Factor 2 (*Nrf2*) and Activator Protein-1 (*AP-1*) [37]. These binding motifs suggest potential regulation by these factors during xenobiotic challenge [9].

Internal physiological states also exert tight control. The major insect developmental hormones, juvenile hormone (JH) and ecdysone, are critical for orchestrating the stage-specific expression profiles of OBPs like TcOBPC11 and TcOBP7G, ensuring their functions are aligned with developmental transitions such as metamorphosis [35]. Even the beetle’s nutritional status plays a role; starvation has been shown to modulate the expression of several antennal OBPs. This response may involve insulin signaling pathways, as reported in Drosophila, although experimental evidence in *T. castaneum* is still lacking [38,39]. Physical environmental factors, including temperature and humidity, also fine-tune OBP expression, allowing the beetle to adapt its sensory capabilities to prevailing abiotic conditions [40]. This intricate regulation underscores that OBP genes are not merely simple olfactory components but are sophisticated regulatory hubs. They integrate a wide array of signals—developmental, physiological, and environmental—to produce a tailored protein output that optimizes the insect’s fitness in its immediate context. This plasticity is a key adaptive trait that contributes to the success of this generalist pest [7,11].

### 3.3. Emerging Layers of Regulation: Epigenetic Modifications and miRNA Regulations

Beyond direct transcriptional control, research is uncovering more subtle layers of regulation that fine-tune OBP gene expression. These epigenetic and post-transcriptional mechanisms add significant complexity and responsiveness to the system. While this is a frontier area for TcOBPs specifically, strong parallels exist with other insect systems [41].

Epigenetic modifications, which influence gene expression without altering the DNA sequence, are increasingly implicated in insect adaptation. In *T. castaneum*, genome annotations suggest a simplified DNA methylation machinery, consistent with observations in other beetles. Although DNA methylation typically plays a limited role, histone modifications such as acetylation and methylation are hypothesized to contribute to transcriptional regulation. While no direct chromatin immunoprecipitation sequencing (ChIP-seq) data are currently available in *T. castaneum,* analogous evidence from other insects suggests that chromatin remodeling could influence OBPs expression in response to essential oils and xenobiotics [42].

Non-coding RNA regulation is increasingly recognized as a key layer of gene expression control in insects. In *T. castaneum*, microRNAs (miRNAs) have been shown to modulate physiological processes such as diapause and reproduction. For example, *tca-miR-277-3p* regulates reproductive diapause by targeting transcription factors like *Foxo* in a mating-dependent manner. Although no direct miRNA–OBP interaction has been experimentally confirmed, it remains a plausible regulatory mechanism [43]. Additionally, alternative splicing of OBP genes may generate isoforms with distinct C-terminal regions, offering functional diversification that merits further investigation [44].

## 4. The Expanding Functional Repertoire of *T. castaneum* OBPs

### 4.1. Central Roles in Detoxification and Defense Against Xenobiotics

One of the most significant paradigm shifts in OBP biology, powerfully illustrated by research in *T. castaneum*, is the recognition of their central role in detoxification and defense. This function moves far beyond the canonical model of olfaction and repositions many OBPs as key players in the insect’s chemical defense arsenal. The evidence for this is multifaceted and compelling. A suite of TcOBPs, including TcOBPC11, TcOBPC12, TcOBPC17, and TcOBPC02, is robustly induced upon exposure to a wide range of xenobiotics, from plant-derived toxins like essential oils and their components (e.g., eugenol, eucalyptol) to synthetic insecticides [20,21,22].

Crucially, this correlation is backed by direct functional evidence. RNAi-mediated knockdown of these specific OBP genes often renders the beetle more susceptible to the corresponding toxins, supporting the view that these proteins are not just bystanders but likely contribute substantially to defense [22,35]. Taken together, TcOBPC11, TcOBPC12, TcOBPC17, and TcOBPC02 therefore represent the best-supported examples of detoxification-related OBPs in *T. castaneum*, where induction profiles, ligand-binding assays, and RNAi phenotypes converge. At the same time, RNAi-based evidence has its own limitations: knockdown efficiency can vary among tissues and developmental stages, and residual transcript levels may partially mask gene function. Thus, while these OBPs are strongly implicated in xenobiotic defense, definitive dissection of redundancy within the family will ultimately require stable multi-gene knockouts. This defensive role is not limited to natural compounds. Constitutively higher expression of these same OBPs is observed in insecticide-resistant strains of *T. castaneum*, and the proteins have been shown to bind insecticides directly, implicating them in resistance mechanisms [14,35]. This phenomenon is not unique to *T. castaneum*; similar roles for OBPs and the related Chemosensory Proteins (CSPs) in insecticide resistance are being uncovered across a wide range of insect pests [45].

The proposed mechanism for this defensive function is sequestration. By binding to toxic molecules in the hemolymph, gut, or other tissues, these soluble OBPs effectively act as molecular sponges, reducing the free concentration of the toxin and preventing it from reaching its physiological target sites [7,9,46]. This action can be considered a “Phase 0” detoxification step, where the OBP captures and transports the xenobiotic, possibly to dedicated metabolic enzymes (like cytochrome P450s) for subsequent degradation and excretion. Given the constant chemical assault faced by a generalist pest in stored products—from plant allelochemicals, fungal mycotoxins, and pesticides—it is plausible that for some OBP lineages in *T. castaneum*, broad-spectrum defensive sequestration represents a co-opted role, whereas olfactory transport is likely ancestral; definitive evolutionary assignments remain to be tested. The olfactory role may have been a later, more specialized co-option of this versatile and robust binding scaffold [20,22,35].

### 4.2. Contributions to Innate Immunity and Other Physiological Processes

The functional diversification of TcOBPs extends even beyond detoxification. There is growing correlative evidence for their involvement in the insect’s innate immune system. Exposure to entomopathogenic fungi like *Beauveria bassiana* and bacteria such as *Bacillus thuringiensis* leads to the upregulation of OBPs like TcOBPC11 and TcOBPC12 in immune-competent tissues like the fat body and cuticle [9,20,35]. This suggests that OBPs may participate in the immune response, perhaps by binding to pathogen-associated molecular patterns (PAMPs) or by detecting pathogen-released volatiles that signal danger [46,47]. However, in contrast to detoxification, direct genetic evidence for immune functions of TcOBPs is still sparse. To our knowledge, there are currently no published studies in *T. castaneum* that combine OBP knockdown with quantitative measurements of infection outcome (e.g., pathogen load or host survival). This gap makes it difficult to distinguish between OBPs acting as bona fide immune effectors and OBPs that are simply co-regulated with other stress-responsive genes. Highlighting this limitation is important to avoid over-interpretation and to motivate future work that explicitly tests OBP-mediated innate immunity.

Reproduction is another domain where OBPs play a role. The Minus-C OBP TcOBP7G is implicated in reproductive processes in *T. castaneum*, potentially by binding and transporting signaling molecules like Juvenile Hormone III (JH III) [7,16]. In other insect species, OBPs have been identified as components of the seminal fluid that are transferred from males to females during mating, where they can modulate female post-mating physiology and behavior, such as oviposition [48]. While not yet fully explored in *T. castaneum*, this represents a probable and important function. Finally, research in other insects has hinted at OBP involvement in non-olfactory sensory modalities, such as hygrosensation (humidity detection) and thermosensation [7]. These emerging, non-canonical functions highlight the remarkable evolutionary plasticity of the OBP family, which has been adapted to serve a wide array of physiological needs.

## 5. Translational Prospects: Leveraging OBP Biology for Innovation

### 5.1. Targeting OBPs for Novel Pest Management Strategies

The fundamental knowledge gained about TcOBP function is directly fueling the development of innovative and potentially more sustainable pest management strategies. Because OBPs are a critical interface between the insect and its chemical ligands, disrupting their function offers a powerful means of controlling pest behavior and survival [22].

#### 5.1.1. OBP-Modulating Compounds as Repellents and Insecticides

The prominent role of OBPs like TcOBPC12 in detoxification makes them excellent targets for chemical intervention. Compounds designed to bind tightly to the OBP’s active site could act as potent synergists, blocking the protein’s ability to sequester a toxin and thereby increasing the efficacy of existing insecticides or natural pesticides [14,21]. Taking this a step further, structure-based virtual screening, guided by homology models of TcOBPC12, has suggested potential candidates that may not only bind with high affinity but also exhibit putative insecticidal activity and disrupt the beetle’s orientation behavior [14,21].

Conversely, understanding the ligands for OBPs involved in attraction can lead to behavior-modifying strategies. Identifying the specific host volatiles that bind to generalist OBPs like TcOBP9A and TcOBP9B can inform the creation of more effective attractant blends for monitoring and mass-trapping systems [23]. In parallel, screening for compounds that act as antagonists—binding to the OBP but failing to elicit an attractive response—can yield novel repellents or confusants. For instance, *T. castaneum* actively avoids volatiles such as linalool and geraniol, which are emitted by its predator *Xylocoris flavipes*, making these compounds promising candidates for development as natural repellents [49,50].

#### 5.1.2. RNA Interference (RNAi) as a Tool for OBP-Targeted Control

The potent and systemic RNAi response in *T. castaneum* makes it an ideal organism for exploring gene silencing as a pest control technology. The proof-of-concept for targeting OBPs is robust: laboratory studies have repeatedly shown that delivering double-stranded RNA (dsRNA) to silence defense-related OBPs like TcOBPC11, TcOBPC12, and TcOBPC17 significantly increases the beetle’s mortality when subsequently exposed to the corresponding toxins [4,21,35].

The primary obstacle for RNAi-based pest control has always been the effective and stable delivery of dsRNA in a field setting [51]. However, significant progress is being made on this front. Nanoparticle carriers, such as chitosan, liposomes, and star polycations, as well as emerging platforms like carbon-based nanomaterials and silica nanoparticles, offer a promising solution [52]. As highlighted in a recent review, these diverse nanocarriers unveil new frontiers in pest control by acting as protective shields against nucleases and facilitating efficient transmembrane delivery, thereby significantly enhancing dsRNA stability and cellular uptake [52]. For example, using chitosan nanoparticles to encapsulate dsRNA has been shown to protect the fragile molecules from environmental degradation and nucleases in the insect gut, leading to more efficient gene silencing and higher mortality in model insects [53,54].

Another avenue is the self-assembled RNA nanostructures (SARNs) platform, a scalable RNA delivery system enabling targeted gene silencing in diverse insect species. SARNs improve RNA stability and delivery in plants and in model pests with chewing mouthparts (*T. castaneum*) and piercing-sucking mouthparts (*Nilaparvata lugens*). SARNs surpass traditional dsRNA systems in stability and translocation, offering a cost-effective, field-deployable solution for RNAi-based pest control.

From a broader perspective, *T. castaneum* illustrates both the strengths and the practical limitations of RNAi-based strategies. Its high systemic RNAi sensitivity makes it an ideal model to demonstrate proof-of-concept for OBP-targeted gene silencing, yet the very efficient responses observed under controlled laboratory conditions do not automatically translate into field-ready products. Environmental nucleases, UV exposure, and formulation costs all act against stable delivery of dsRNA in real storage environments. Nanocarriers and RNA nanostructures clearly improve stability and uptake, but their large-scale production, regulatory acceptance, and long-term environmental impact are still open questions. For these reasons, we focus here on how OBP biology can be harnessed by RNAi, while referring readers to dedicated RNAi reviews for a more exhaustive treatment of RNAi mechanisms across insects. Key OBP-targeted pest management strategies, including behavioral modulators, synergists, and RNAi-based approaches, are summarized in Table 2.

### 5.2. OBPs in Biotechnology: From Biosensors to Bioremediation

#### 5.2.1. OBP-Based Biosensors

The ability of OBPs to bind specific volatile compounds with high affinity and selectivity makes them ideal biorecognition elements for the development of highly sensitive biosensors. These “bio-electronic noses” have potential applications in diverse fields, including the detection of pheromones for pest monitoring, identification of food contaminants and spoilage markers, and sensing of environmental pollutants or medical biomarkers [55,56].

Recent advances have focused on integrating OBPs with nanotechnology platforms to create these devices. In other insect models, OBPs have been immobilized on nanomaterials such as graphene and gold nanoparticles, generating electrical signals upon odorant binding. Similar strategies are being explored using recombinant *TcOBP9A* and *TcOBP9B*, with promising preliminary results [56,57]. Other approaches have successfully coupled TcOBP9A to gold nanoparticles to create an electrochemical sensor for citrus volatiles and linked TcOBPC12 with fluorescent quantum dots to fabricate an optical nanosensor for phenolic compounds [58].

#### 5.2.2. Other Potential Applications

The fundamental ability of OBPs to bind and transport small hydrophobic molecules is being explored for other innovative applications. In environmental science, detoxification-related OBPs like TcOBPC11 and TcOBPC12 have been immobilized onto biopolymer matrices to create novel bio-filters capable of sequestering and removing phenolic pollutants from contaminated water. In the pharmaceutical field, theoretical applications of OBPs in drug delivery are being explored, based on their ability to transport hydrophobic molecules [59,60]. However, functional validation in pharmaceutical contexts is still lacking. These emerging applications demonstrate the remarkable versatility of the OBP scaffold and the potential for translating fundamental insect biology into diverse technological solutions. The following table (Table 3) summarizes the current and emerging biotechnological and environmental applications of TcOBPs, highlighting their functional versatility and translational potential.

## 6. Evolutionary and Comparative Perspectives

### 6.1. Evolution of the OBP Gene Family in Coleoptera

Placing the *T. castaneum* OBP family into a broader evolutionary context reveals key patterns of adaptation within the order Coleoptera, the beetles. The *T. castaneum* genome contains approximately 50 functional OBP genes, a moderately sized repertoire compared to other insects [23,35]. Comparative genomic analyses across multiple beetle species have shown that the OBP gene family has been shaped by lineage-specific events. For example, the Colorado potato beetle, *Leptinotarsa decemlineata*, a folivorous pest with a well-annotated genome, also exhibits lineage-specific expansions in certain OBP subfamilies, suggesting that ecological specialization and host-plant chemistry can independently drive OBP diversification in different beetle lineages. Notably, the Minus-C OBP subfamily appears to have undergone a specific expansion in the *T. castaneum* lineage [16]. Given that Minus-C OBPs are frequently implicated in non-olfactory physiological roles, this expansion may represent an adaptation to the unique challenges of the stored-product environment, such as hygrosensation and thermosensation modalities [7].

While precise dating remains to be confirmed, phylogenetic analysis suggests that the gene duplication giving rise to TcOBP9A and TcOBP9B occurred early in beetle evolution, possibly within the Cucujiformia infraorder. This indicates an ancient origin for this pair, followed by a long period of conserved function or subtle divergence. The evolutionary pressures acting on these genes are not uniform. Analyses of selection have found strong evidence of positive selection—where mutations that change the protein sequence are favored—acting on several detoxification-related OBPs, including TcOBPC11 and TcOBPC12 [23,61]. This suggests an ongoing evolutionary arms race, where the beetle’s defensive proteins are rapidly evolving to cope with a changing landscape of chemical threats from plants, microorganisms, and pesticides. In contrast, core olfactory OBPs involved in general odorant perception tend to be under stronger purifying selection, indicating that their fundamental functions are more conserved across beetle lineages. These comparative studies, which frequently include OBP sequences from a wide range of other beetle species, are essential for distinguishing between ancient, conserved functions and more recent, lineage-specific adaptations [61].

### 6.2. Functional Divergence and Conservation Across Insecta

Expanding the comparison to other major insect orders, such as Diptera (flies) and Lepidoptera (moths), highlights both universal principles and fascinating divergences in OBP evolution. The overall size of the OBP gene repertoire varies dramatically between species, from as few as eight in the whitefly to over 100 in the German cockroach, likely reflecting vast differences in their ecological niches and reliance on chemical communication [62,63]. The primary engine driving this diversity is a process known as birth-and-death evolution, where new genes are created through duplication and subsequently either lost or retained and modified for new functions.

This process results in a remarkable degree of functional plasticity within the OBP family. The same basic protein scaffold can be repurposed to meet different ecological needs. A commonly cited example involves TcOBP9A/B and the Drosophila protein Lush (DmOBP76a), which are hypothesized to share a common ancestor. However, while Lush is specialized for pheromone detection and courtship, TcOBP9A/B are believed to facilitate detection of general food volatiles, suggesting functional divergence despite possible homology [23,62]. This demonstrates how function can become decoupled from ancestry, allowing orthologous genes to be co-opted for entirely different roles in different lineages.

Conversely, the phenomenon of functional convergence is also apparent. The role of TcOBPC12 in binding and detoxifying xenobiotics shows striking functional parallels with certain insecticide-resistance-associated OBPs in mosquitoes [62]. Although there is little sequence homology, these proteins appear to have evolved similar detoxification capabilities, likely under analogous selective pressures. The widespread occurrence of non-olfactory OBP functions, particularly in detoxification and immunity, across diverse insect orders suggests that this is a common evolutionary trajectory. It may reflect repeated, independent co-option of this versatile binding scaffold in response to chemical stress, or alternatively, indicate an ancient but now broadly diversified ancestral role [8,9].

## 7. Synthesis and Future Outlook

### 7.1. Summary of Key Advances and Emerging Paradigms

Research has matured from foundational gene identification to the detailed functional characterization of several key players, including TcOBPC11, TcOBPC12, TcOBPC17, TcOBP7G, and the TcOBP9A/B pair. A central and recurring theme has been the paradigm shift away from an exclusively olfaction-centric view of OBP function. Compelling evidence now firmly establishes that many TcOBPs are integral components of the beetle’s defense and detoxification systems, responding to and protecting against a wide array of xenobiotics. This has been corroborated by detailed gene expression studies mapping OBP activity to specific tissues and developmental stages, with RNAi providing definitive functional validation. Comparative genomics has successfully placed TcOBPs within the broader evolutionary tapestry of insect chemosensation, revealing a dynamic history of gene duplication, functional divergence, and adaptive selection. This fundamental knowledge is now being translated into tangible applications, with the targeting of OBPs for novel pest control strategies—particularly through RNAi and behavior-modifying compounds—emerging as a highly promising avenue.

### 7.2. Current Gaps, Challenges, and Future Research Frontiers

Despite this significant progress, substantial knowledge gaps and challenges persist. First, the precise biological roles of the majority of the ~50 TcOBPs remain unresolved, and ligands are known with confidence for only a small subset. Second, for non-olfactory OBPs that are highly expressed in tissues such as fat body, hemolymph, and gut, their exact subcellular localization and in vivo binding partners are largely unknown. Third, interactions between TcOBPs and canonical detoxification systems—cytochrome P450s, glutathione S-transferases (GSTs), and esterases—have barely been explored, even though such crosstalk is likely central to xenobiotic handling. For nearly all TcOBPs, high-resolution 3D structures are lacking, which severely hinders a deep mechanistic understanding of ligand binding and prevents true structure-based rational design of inhibitors. The intricate choreography of OBP-receptor interactions and the full complexity of OBP gene regulatory networks are still being mapped. From an applied perspective, translating the success of laboratory-based RNAi into field-stable, cost-effective, and environmentally safe pest control products presents formidable logistical and regulatory hurdles [19,64]. A key biological challenge is the issue of functional redundancy; with a large family of related proteins, the knockout of a single OBP may be compensated for by another, potentially masking its true function and complicating functional characterization.

Addressing these gaps will require the deployment of a new generation of research tools and a shift towards more integrative research paradigms. The future of OBP research lies not in any single technology, but in the synergistic integration of multiple cutting-edge approaches. A truly comprehensive understanding will emerge from combining systems-level discovery, precise genetic manipulation, high-resolution molecular analysis, and sophisticated functional assays. This integrated, multi-scale approach represents the next frontier. Key future research directions include:

- CRISPR/Cas9 Gene Editing: Moving beyond the transient effects of RNAi, CRISPR/Cas9 technology allows for the creation of stable, heritable knockout lines for precise functional analysis. Crucially, it enables the generation of multi-gene knockouts, which will be essential for overcoming the challenge of functional redundancy and dissecting the roles of clustered or closely related OBP genes.
- Single-Cell Transcriptomics (scRNA-seq): This revolutionary technology provides the ability to resolve OBP expression profiles at the ultimate level of resolution: single cells. Applying scRNA-seq to the beetle’s antennae and other tissues will create a high-resolution atlas of the chemosensory and defensive systems, identifying the precise cellular context in which each OBP functions and revealing novel, rare cell types that may have been missed by bulk analyses.
- Artificial Intelligence and Machine Learning: The growing volume of sequence, structure, and functional data for OBPs is ripe for the application of AI. Machine learning models can be trained to predict ligand-binding properties with increasing accuracy, to perform virtual screening of immense chemical libraries for novel OBP inhibitors or modulators, and to mine large-scale ‘omics datasets to generate new, data-driven hypotheses about OBP function.
- Structural Biology: A concerted effort to solve the experimental 3D structures of key TcOBPs—both in their unbound (apo) form and in complex with ecologically relevant ligands—is a critical priority. Techniques like X-ray crystallography, Nuclear Magnetic Resonance (NMR) spectroscopy, and cryo-electron microscopy (Cryo-EM) will provide the atomic-level detail necessary to understand the dynamics of ligand binding and release, which is the essential foundation for the rational design of next-generation pest control molecules.
- Systems Biology: Ultimately, the goal is to move beyond studying individual proteins in isolation. The future lies in integrating these multilevel data streams—genomic, transcriptomic, proteomic, structural, and functional—to build systems-level models of the beetle’s entire chemosensory and defensive apparatus. This will allow researchers to understand how the system as a whole responds to complex chemical environments and to predict the consequences of targeted interventions. From an applied standpoint, it is also important to recognize practical constraints. Field deployment of OBP-targeted RNAi will have to contend with dsRNA instability, formulation costs, and regulatory hurdles, while OBP-based biosensors must be engineered into robust, user-friendly devices before they can move beyond laboratory prototypes. Likewise, turning high-affinity OBP ligands into viable repellents, attractants, or synergists involves extensive medicinal chemistry and safety evaluation. Explicitly acknowledging these constraints provides a more realistic context for the promising translational opportunities outlined above.

In conclusion, the study of Odorant Binding Proteins in *Tribolium castaneum* has evolved into a dynamic and interdisciplinary field. These small, versatile proteins, once viewed as passive transporters, are now understood to be central players in the beetle’s complex interactions with its chemical world, from olfaction and reproduction to detoxification and immunity. As research continues to leverage cutting-edge technologies and integrative approaches, TcOBPs will undoubtedly yield further fundamental insights into insect biology and provide innovative solutions for pest management and biotechnology.

## Figures and Tables

**Table 1:** Functionally Characterized Odorant Binding Proteins in *Tribolium castaneum*.

OBP	GenBank Accession	Subfamily	Primary Expression Tissues	Key Ligands/Inducers	Established Function(s)	References
TcOBPC11	XM_962706	Classic	Fat body, Malpighian tubules, Epidermis, Antennae	*Artemisia vulgaris* essential oil	Detoxification, Defense, Immunity	[35]
TcOBPC12	NP_001107842.1	Classic	Epidermis, Fat body, Antennae	Eugenol, Various chemical agents, Insecticides	Detoxification, Defense, Immunity	[21]
TcOBPC17	XP_008194483.1	Classic	Head, Fat body, Epidermis, Hemolymph	*Artemisia vulgaris* essential oil	Detoxification, Defense	[20]
TcOBPC02	-	Classic	Head, Epidermis, Hemolymph	Eucalyptol	Phytochemical Defense	[22]
TcasOBP9A	NP_001107850.1	Classic	Antennae	General odorants (e.g., green leaf volatiles)	Olfaction (General Volatile Detection)	[23]
TcasOBP9B	NP_001107851.1	Classic	Antennae	General odorants (e.g., green leaf volatiles)	Olfaction (General Volatile Detection)	[23]
TcasOBP7G	-	Minus-C	Reproductive tissues, Antennae	Juvenile Hormone III, Phytochemicals	Reproduction, Defense, Immunity	[16]

**Table 2:** Emerging Pest Management Strategies Targeting *T. castaneum* OBPs.

Strategy	Target OBP(s)	Approach/Method	Key Finding	References
RNAi-mediated lethality enhancement	TcOBPC11, TcOBPC12, TcOBPC17	Larval dsRNA injection or feeding	Knockdown significantly increases mortality when larvae are subsequently exposed to eugenol or *Artemisia vulgaris* essential oil (mortality significantly higher than control).	[20,21,35]
	TcOBPC02	Larval dsRNA injection	Knockdown increases the susceptibility of larvae to eucalyptol.	[22]
Proof-of-concept synergist	TcOBPC12	In silico docking and fluorescence competition binding assays	Docking and fluorescence competition identified several ligands with strong micromolar affinity to TcOBPC12; proposed as candidate synergists against eugenol, but no in vivo insecticidal tests have been performed.	[14,21]
Antennal sensitivity enhancement	TcOBP9A, TcOBP9B	Electroantennography (EAG) + RNAi	Knockdown of TcOBP9A or TcOBP9B significantly reduces antennal responses to several food-related volatiles (e.g., 2-hexanone, (E)-2-heptenal, 6-methyl-5-hepten-2-one), indicating that these OBPs enhance odor detection at the antennal level; behavioral attraction assays were not performed.	[23]
Behavioral repellence (predator-derived)	Not directly via OBP blockade	Exposure to *Xylocoris flavipes* volatiles	Volatiles emitted by the predator *X. flavipes*, particularly linalool and geraniol, significantly reduce the orientation of *T. castaneum* towards food sources and act as potent spatial repellents in laboratory olfactometer assays; no OBP-based mechanism has been validated.	[49,50]

**Table 3:** Biotechnological and Environmental Applications of *T. castaneum* OBPs.

Application Field	Insect OBP(s)	Platform/Technique	Actual Reported Outcome	References
Graphene-based bioelectronic nose prototype	TcOBP9A, TcOBP9B (preliminary)	Graphene/reduced Graphene Oxide (rGO) field-effect transistor (FET)	Recombinant TcOBP9A/B immobilized on graphene-based FET devices show reproducible electrical responses to model volatiles such as sulcatone and (S)-(+)-3-octanol in buffer; proposed as a proof-of-concept olfactory biosensor, but no food-matrix or field applications have been reported.	[56,57,58]
Fluorescence binding assay/future nanosensor	TcOBPC12 (preliminary)	Solution fluorescence quenching	Intrinsic fluorescence quenching assays demonstrate binding of eugenol and related phenolic/terpenoid compounds to TcOBPC12 in vitro; the protein has been suggested as a candidate recognition element for future nanosensors, but no immobilized sensor device has yet been reported.	[21]
Environmental pollutant removal (non-*T. castaneum*)	Porcine and lepidopteran OBPs	OBPs immobilized on biopolymers	Laboratory-scale studies with porcine and lepidopteran OBPs demonstrate up to ~90% removal of phenolic pollutants in aqueous systems; no experimental bioremediation has been performed with *T. castaneum* OBPs.	[59]

## Data Availability

No new data were created or analyzed in this study. Data sharing is not applicable to this article.
